# Impact of the BHOF FLS TeleECHO program: a review of role and outcomes

**DOI:** 10.1007/s11657-025-01557-w

**Published:** 2025-06-11

**Authors:** Ami R. Patel, Nora Laberee, Andrea P. Medeiros

**Affiliations:** 1 Bone Health & Osteoporosis Foundation, 251 18th Street S, Suite 630, Arlington, VA 22202 USA; 2 University of Michigan School of Public Health, 1415 Washington Heights, Ann Arbor, MI 48109 USA

**Keywords:** Osteoporosis, Fracture liaison service, TeleECHO, Bone health, Secondary fracture prevention

## Abstract

**Introduction:**

Osteoporosis-related fractures represent a major public health issue, impacting approximately 2 million Americans each year. Notably, 25–30% of these individuals suffer a subsequent fracture within 5 years. The concept of imminent fracture risk, which highlights the elevated risk of fracture within 1–2 years following an initial event, emphasizes the urgency of secondary prevention strategies. Fracture Liaison Service (FLS) programs have been shown to reduce the risk of subsequent fractures by up to 50%.

**Methods:**

This review evaluates the Bone Health and Osteoporosis Foundation (BHOF) FLS ECHO program, which employs a telementoring model to guide and support healthcare providers in establishing and enhancing FLS programs. Data were collected from participant surveys to assess satisfaction and the application of knowledge gained through the program.

**Results:**

Survey responses indicated high levels of participant satisfaction with the FLS ECHO program. Respondents reported a strong commitment to implementing the strategies learned during the sessions to improve secondary fracture prevention within their practices.

**Conclusion:**

The BHOF FLS ECHO program shows promise in addressing the burden of osteoporotic fractures by increasing the adoption and effectiveness of FLS models. Through targeted education and virtual mentorship, the program can expand access to secondary fracture prevention strategies, ultimately improving patient outcomes.

## Introduction

Osteoporosis, characterized by low bone mineral density (BMD) and increased bone weakness, affects nearly 20% of women and 5% of men over age 50 [1, 2].

Osteoporosis-related fractures represent a major public health burden, with approximately 2 million fractures occurring annually in the United States alone, resulting in over $19 billion in related healthcare costs [1, 2]. The risk of subsequent fracture is substantially elevated following an initial fracture, with studies showing that individuals who experience a fragility fracture face a two to four times increased risk of subsequent fracture compared to those without prior fractures [3, 4]. Moreover, approximately 25–30% of patients will experience a second fracture within 5 years of their initial fracture, with the highest risk occurring within the first 1–2 years—a phenomenon known as imminent fracture risk [4].

The severe consequences of these fractures, including significant pain, disability, decreased function, and high morbidity and mortality, have made this a key area of focus for researchers and healthcare providers [3]. Mortality rates following hip fractures can reach 20–30% within the first year [1, 4], further emphasizing the critical need for effective prevention strategies.

In recent years, the undertreatment of patients with osteoporosis-related fractures has become a substantial concern, with differences in treatment guidelines and ambiguity around optimal patient care contributing to this gap in care. Studies show that less than 20% of patients with fragility fractures receive appropriate osteoporosis evaluation and treatment, creating a significant care gap. Post Fracture Care (PFC) programs, particularly the fracture liaison service (FLS) model, have been established to provide secondary fracture prevention services and improve the management of osteoporosis [3].

The FLS program, first described in 2003 by McLellan et al. [5], has been replicated worldwide with substantial success. These programs typically include components of patient monitoring and assessment, osteoporosis treatment, and fall prevention and education services [3]. Studies have shown that FLS programs improve outcomes for patients and a reduction in the risk of further fractures by up to 30% [6] significant reductions in re-fracture and mortality rates [7].

The financial burden of osteoporosis-related fractures is significant, with current costs associated with hip fractures in the US exceeding $10 billion per year [8]. Studies have demonstrated the cost-saving potential of FLS programs, with one analysis calculating potential savings of approximately $418 million for every 1 million patients in the US Medicare system [9].

This review focuses on the Bone Health and Osteoporosis Foundation (BHOF) FLS ECHO program, launched in 2017, which aims to support healthcare providers in launching and growing FLS programs within their communities to address the critical need for improved secondary fracture prevention.

## Methods

### Program collection

The BHOF FLS ECHO program utilizes the Project ECHO (Extension for Community Healthcare Outcomes) model, a revolutionary telementoring approach designed to connect healthcare providers and promote education and development [10, 11]. Unlike traditional telemedicine or webinar platforms, the ECHO model facilitates interactive, case-based learning where all participants both learn and teach, creating a virtual community of practice.

The program involves regular monthly video conferences where healthcare providers present real, de-identified patient cases for collaborative discussion. This format allows for the dissemination of best practices and expert knowledge to providers in various settings, including rural and underserved areas. By fostering an “all teach, all learn” environment, the program helps build capacity and confidence among healthcare providers in implementing effective FLS programs.

### Data collection

The BHOF conducted a survey research project to assess the efficacy and impact of the FLS ECHO program. Participants were surveyed following each monthly session from June 2023 to April 2024, excluding July, September, November, and March due to session cancellations. Surveys were administered using the EthosCE learning management system.

Survey questions included demographic and career information, session-specific questions, and 11 questions regarding survey quality and impact that remained consistent across all session surveys. The 7 months of data produced 24 survey responses from unique participants across the US. Results were analyzed using Microsoft Excel.

Additional data from 21 responses collected in 2021 over 4 months of surveys administered through CME360 by Premier were also considered to provide further insight into participants’ opinions of the program and its impact on their actions.

Geographic distribution data and institutional participation information were collected through program registration records maintained by BHOF from 2018 to 2024 in the learning management systems. These records were analyzed to identify the locations of participating healthcare institutions and their distribution across states.

## Results

**Fig. 1:**
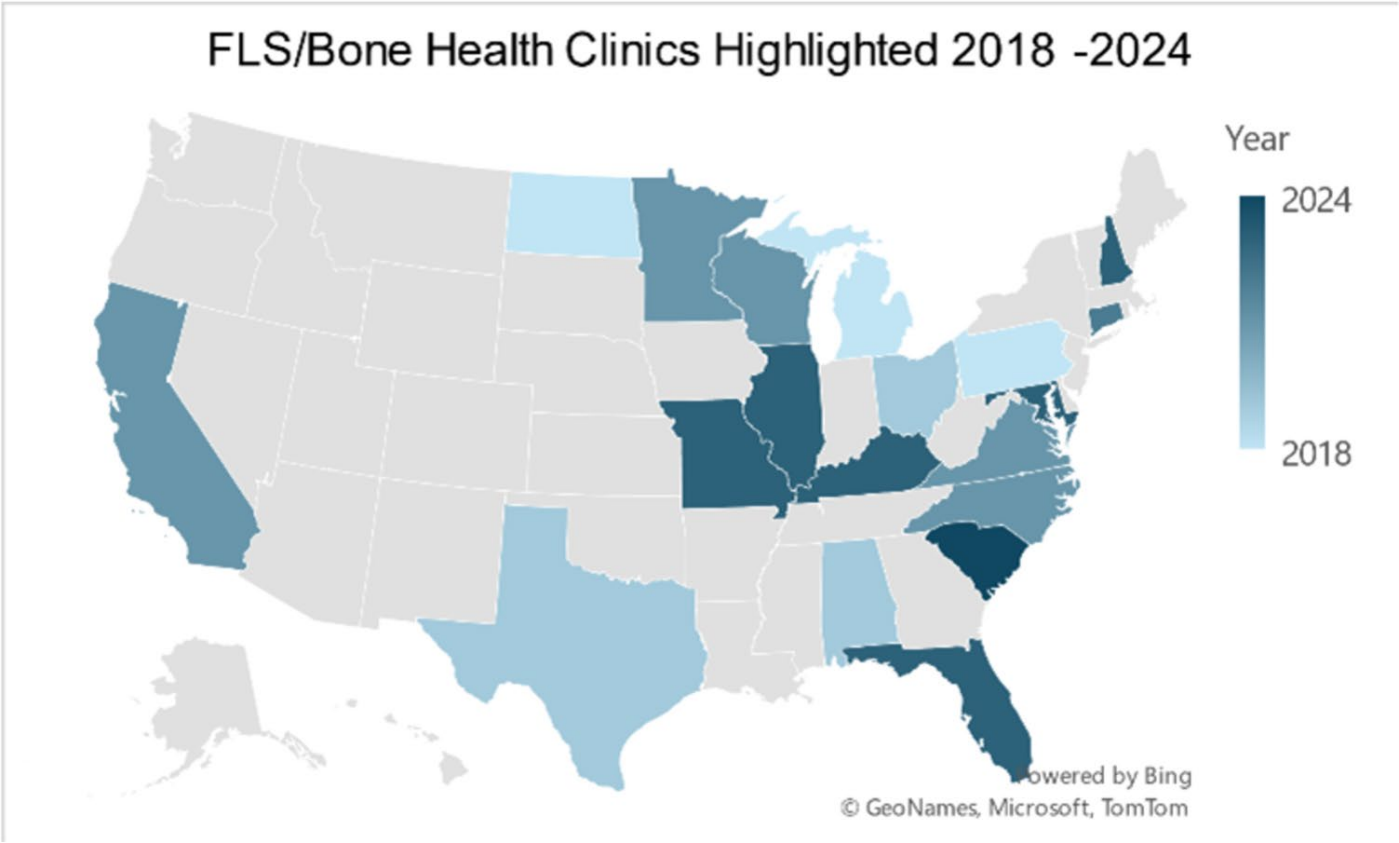
Geographic distribution of FLS ECHO program participating institutions in the United States, 2018–2024. Map illustrates the locations of 32 healthcare institutions that have participated in the BHOF FLS ECHO program across 24 states

The FLS ECHO program has established a comprehensive national footprint, delivering presentations through 32 healthcare institutions across 24 states (as shown in Fig. 1) from 2018 to 2024. The program’s reach extends through a diverse network of healthcare providers, encompassing major academic medical centers, regional health systems, and specialized orthopedic practices. This broad institutional participation demonstrates the program’s successful adaptation to various healthcare settings and service delivery models. The program has maintained consistent growth while effectively serving both urban and rural communities, supporting its mission to expand access to bone health expertise across diverse geographical and clinical environments.

**Fig. 2:**
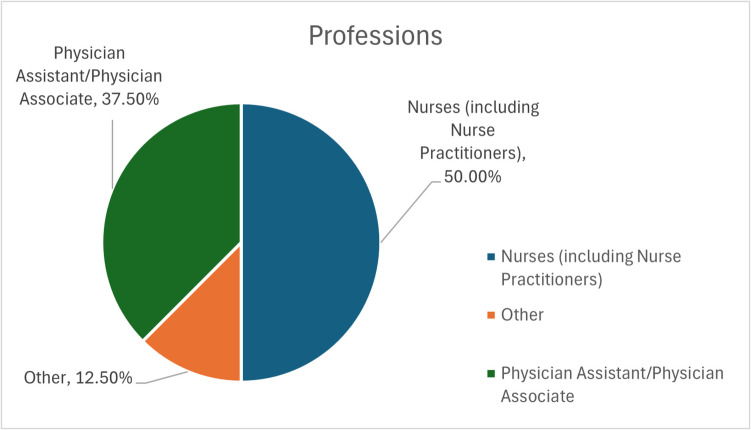
Professions of FLS participants. Chart shows the distribution of professions among survey respondents, with 50% nurses and nurse practitioners, 38% physician assistants, and 12% other professions including orthopedic NPs and physical therapists

**Fig. 3:**
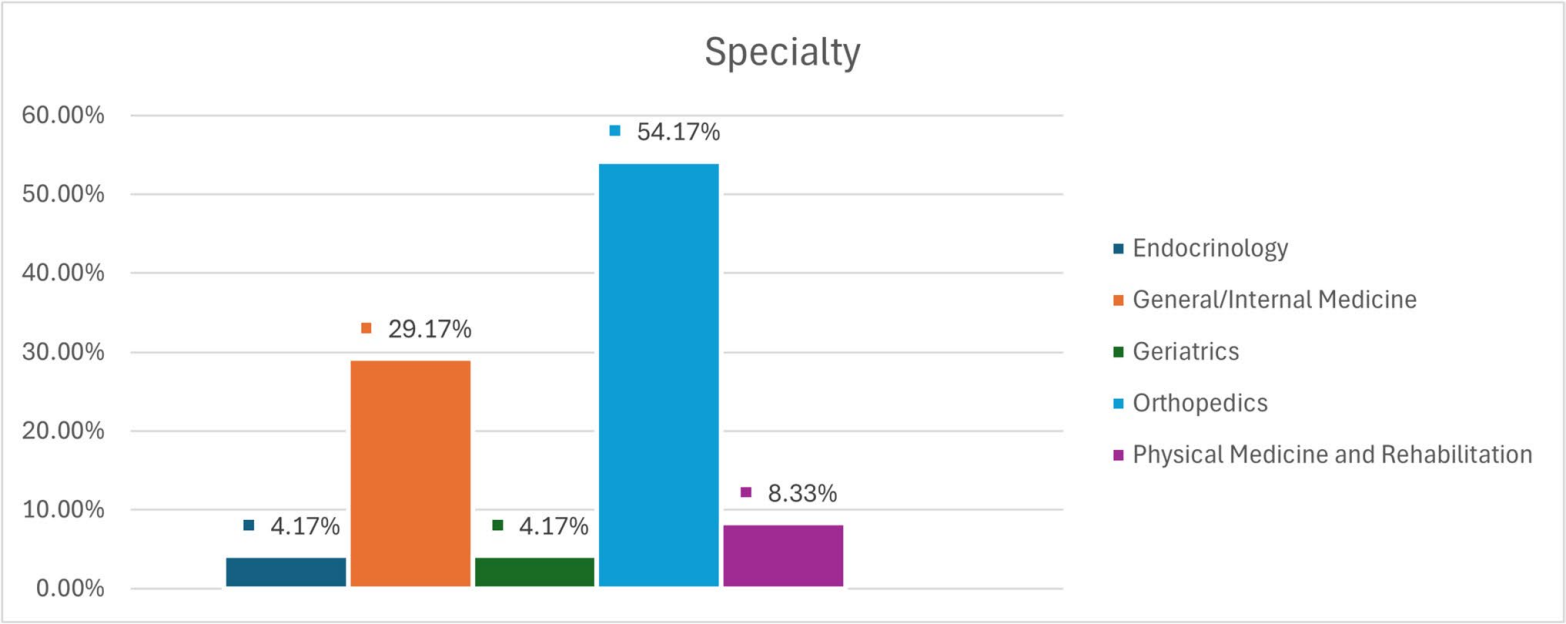
Specialty of FLS participants. Chart illustrates the distribution of specialties among survey respondents, with 54% in orthopedics, 29% in general/internal medicine, 9% in physical medicine and rehabilitation, 4% in geriatrics, and 4% in endocrinology

The BHOF FLS ECHO program survey revealed a diverse participation across healthcare professions and specialties. As illustrated in Fig. 2, nurses constituted the largest group of participants (38%), followed by physicians (25%) and nurse practitioners (12%), with representation from physician assistants (13%), pharmacists (8%), and physical therapists (4%). Figure 3 demonstrates the wide range of medical specialties involved, with orthopedics being the most prevalent (54%), followed by general/internal medicine (29%), physical medicine and rehabilitation (9%), geriatrics (4%), and endocrinology (4%).

Participants represented various practice settings, including multispecialty groups, specialty groups, hospital-based practices, and academic medical centers or universities, indicating the program’s broad reach across different healthcare delivery models. These demographic data were consistent across both the EthosCE surveys and the CME360 surveys conducted in 2021.

**Fig. 4:**
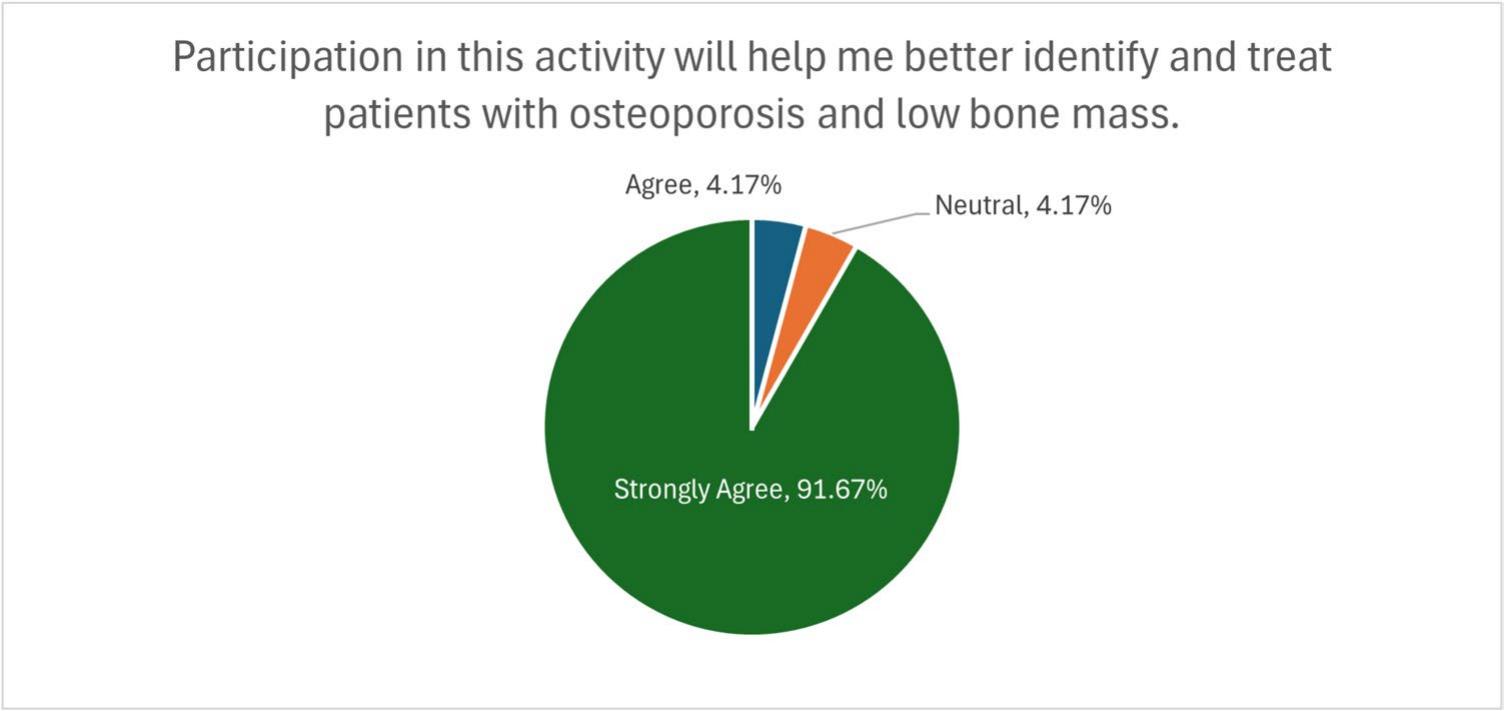
Participants’ ability to better identify and treat patients with osteoporosis and low bone mass. Chart shows the percentage of participants who agreed or strongly agreed that the FLS ECHO program would help them better identify and treat patients with osteoporosis and low bone mass

The survey results demonstrated a highly positive reception of the FLS ECHO program. Notably, 92% of participants agreed or strongly agreed that the program would enhance their ability to identify and treat patients with osteoporosis and low bone mass (Fig. 4). An equal percentage (92%) concurred that the material presented promoted improvements and/or quality in healthcare. These findings underscore the program’s effectiveness in meeting its educational objectives and its potential to enhance the standard of care for patients at risk of fragility fractures.

**Fig. 5:**
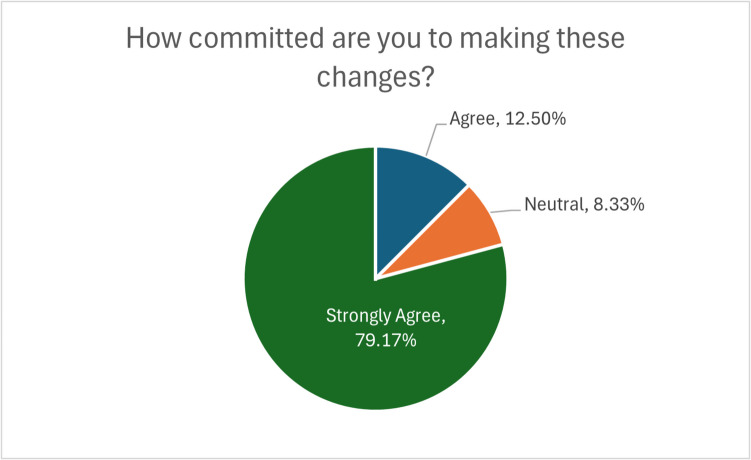
Participants’ commitment to making changes following FLS sessions. Chart displays the levels of commitment among participants to implement changes discussed in the program, with 79% very committed, 13% somewhat committed, and 8% not expecting their practice to change

Participants’ commitment to implementing changes based on the knowledge gained from the FLS ECHO program was particularly encouraging. As shown in Fig. 5, 79% of participants reported being very committed to making changes discussed in the program, with an additional 13% somewhat committed. Only 8% did not anticipate changes in their practice. This high level of commitment suggests that the program is not only imparting knowledge but also motivating healthcare providers to take action to improve patient care.

Participants’ commitment to implementing changes based on the knowledge gained from the FLS ECHO program was particularly encouraging. As shown in Fig. 5, 79% of participants reported being very committed to making changes discussed in the program, with an additional 13% somewhat committed. Only 8% did not anticipate changes in their practice. This high level of commitment suggests that the program is not only imparting knowledge but also motivating healthcare providers to take action to improve patient care.

The 2021 CME360 survey data specifically revealed that participants planned to implement several concrete changes, including increasing pharmacist involvement in their FLS programs, improving understanding of the relationship between type 2 diabetes and osteoporosis, updating knowledge about the impact of calcium and vitamin D on osteoporosis outcomes, enhancing patient education strategies, and gaining better logistical knowledge about hospital operations relating to FLS programs.

These results collectively demonstrate the FLS ECHO program’s effectiveness in reaching a diverse audience of healthcare providers, improving their knowledge and skills related to osteoporosis care, and motivating them to implement positive changes in their clinical practices. The high levels of agreement on the program’s benefits and the strong commitment to practice changes suggest that the FLS ECHO model has significant potential to drive meaningful improvements in osteoporosis care and fracture prevention.

## Discussion

The BHOF FLS ECHO program has demonstrated significant promise in addressing the critical need for improved secondary fracture prevention through expanding the implementation of effective FLS programs. The survey results indicate that this telementoring approach is successful in reaching diverse healthcare providers across multiple specialties and practice settings, suggesting its potential to bridge gaps in care, particularly in areas with limited access to specialty services.

The global landscape of Bone Health ECHO programs has expanded significantly since the inception of the first program in Galway, Ireland in 2014. Currently, there are 19 known Bone Health ECHO programs operating across multiple continents, with the majority (10) based in the US. Programs are also established in Ireland, Russia, Lebanon, Australia/New Zealand, Argentina, and Peru, demonstrating the model’s adaptability to various healthcare systems.

The linguistic landscape of these programs is predominantly English (14 programs), with additional programs in Russian (2) and Spanish (3). This linguistic expansion is crucial for broadening the reach and impact of bone health education and best practices to address the global burden of osteoporosis-related fractures.

The FLS ECHO program’s high satisfaction rates and participants’ strong commitment to implementing changes in practice suggest that this model effectively addresses key barriers to FLS implementation, including knowledge gaps and resource limitations. By creating a virtual community of practice, the program facilitates the sharing of expertise and best practices that can be adapted to diverse clinical settings.

When compared with other Project ECHO initiatives, the BHOF FLS ECHO program shows similar patterns of engagement and participant satisfaction to those reported by Agley et al. [11] in their qualitative findings across five different ECHO programs. Their study highlighted the value of the “all teach, all learn” model in creating communities of practice, which aligns with our program’s approach. Additionally, the Rare Bone Disease TeleECHO program described by Tosi et al. [10] offers a comparable model within the bone health field, though focusing on rare diseases rather than osteoporosis and fracture prevention. Both programs demonstrate the effectiveness of telementoring in improving provider confidence and knowledge in specialized areas of bone health.

The potential implications of expanding FLS ECHO programs globally are substantial. Breaking down language and geographic barriers can democratize access to expertise, potentially improving patient outcomes across diverse contexts. For countries with limited resources, these programs offer a cost-effective method to enhance provider skills without requiring extensive travel or in-person training.

To maximize the global impact of FLS ECHO programs, increasing language diversity, targeting underrepresented regions, and collaborating with local medical societies will be essential. Furthermore, developing a global FLS ECHO network could enhance knowledge sharing across programs, while comprehensive outcome studies will be crucial for demonstrating value and guiding future expansions.

The BHOF FLS ECHO program’s effectiveness in motivating practice changes aligns with the urgent need to address the care gap in osteoporosis management following fragility fractures. Given that less than 20% of fracture patients receive appropriate evaluation and treatment, interventions that successfully increase provider knowledge and motivation to implement FLS programs have significant public health implications.

## Conclusion

Osteoporosis-related fractures represent a growing public health crisis that requires action at multiple levels of the healthcare system. With approximately 25–30% of patients experiencing a subsequent fracture within 5 years of their initial fracture, effective secondary prevention strategies are critical. FLS programs have demonstrated significant and clinically relevant benefits including 30% relative reduction in the risk of further fractures with FLS care vs. non-FLS care [6] and approximately 3% reduction in mortality as reported by [7].

The BHOF FLS ECHO program shows promise in expanding the reach of FLS programs nationwide, potentially helping to address the fracture crisis in areas with limited access to specialty services. The program’s success in engaging diverse healthcare providers and motivating practice changes suggests its potential for significant impact on improving post-fracture care.

Continuing to gather feedback on the efficacy and value of these programs for participants will allow for more targeted and comprehensive design in the future. As the healthcare landscape evolves, telementoring programs like FLS ECHO may play an increasingly important role in disseminating best practices and supporting healthcare providers in implementing effective osteoporosis care strategies to reduce the burden of subsequent fractures.
